# Outcome and Predisposing Factors for Intracranial Hemorrhage in Turkish Children with Hemophilia

**DOI:** 10.3390/jcm14030689

**Published:** 2025-01-22

**Authors:** Defne Ay Tuncel, Hatice İlgen Şaşmaz, Bülent Antmen

**Affiliations:** 1 Department of Pediatric Hematology/Oncology and Bone Marrow Transplantation Unit, University of Health Sciences, Adana City Education and Research Hospital, 01100 Adana, Turkey; 2 Department of Pediatric Hematology, Faculty of Medicine, Balcali Hospital, Cukurova University, 01110 Adana, Turkey; ilgen@cu.edu.tr; 3 Department of Pediatric Hematology/Oncology and Bone Marrow Transplantation Unit, Faculty of Medicine, Adana Hospital, Acibadem University, 01100 Adana, Turkey; bulent.antmen@acibadem.com.tr

**Keywords:** childhood hemophilia, intracranial hemorrhage, pediatric population, mortality rate

## Abstract

**Background/Objectives:** Childhood hemophilia, a hereditary bleeding disorder predominantly affecting males, arises due to gene mutations encoding clotting factors VIII or IX. Intracranial hemorrhage represents a significant and life-threatening complication in pediatric patients with hemophilia. The incidence of intracranial hemorrhage in children with hemophilia, although relatively low, is notably higher compared to the general pediatric population. **Methods**: In this study, the objective is to examine patients with hemophilia who have experienced intracranial hemorrhage retrospectively. This study is a multicenter, retrospective analysis using data from three tertiary care centers in a provincial city in Turkey. Data were obtained from the participants’ hospital records. The presence of inhibitors against FVIII in the participants and the prophylaxis used against them were included in the analysis. Trauma history was queried, with types of traumas examined, including traffic accidents, falls, and a traumatic vaginal delivery. The duration and causes of complaints among the participants were investigated. The causes of complaints were categorized as fever, hematoma, convulsions, loss of consciousness, and hemiparesis. The participants’ Physical Examination Findings were classified as fever, hematoma, and loss of consciousness. The duration of hospital stays was evaluated. The hemorrhage location was classified into five groups: parenchymal, subdural, scalp, subarachnoid, and multiple hemorrhagic foci. The recurrence of bleeding, the need for transfusion, surgical intervention, and mortality were also examined. **Results**: A significant difference was identified between the participants’ survival rates and age variables, as well as transfusion in <36 months. A total of 9 participants had spontaneous intracranial bleeding, 2 experienced cranial trauma as a result of traffic accidents, and 25 participants were exposed to head trauma due to falls. Of the remaining individuals, one suffered head trauma from a severe impact, and one had cranial trauma following a traumatic vaginal delivery. Fourteen participants required transfusion, and three underwent surgical intervention. **Conclusions**: According to the results of the statistical analyses, the variables Factor Level, Physical Examination Findings, Transfusion, Recurrent Bleeding, Inhibitor, and Prophylaxis were found to affect survival significantly. No significant relationship was determined between the other analyzed variables and survival. During our study, five of the participants examined died. Accordingly, the mortality rate identified in our study is 13.1%.

## 1. Introduction

Childhood hemophilia, a hereditary bleeding disorder predominantly affecting males, arises due to mutations in the genes encoding clotting factors VIII (Hemophilia A) or IX (Hemophilia B) [1]. This X-linked recessive disorder manifests early in life, often with prolonged bleeding episodes following minor injuries or surgical procedures [2]. In severe cases, spontaneous hemarthroses, or bleeding into joints, are common, leading to significant morbidity due to joint damage and chronic pain [3].

Intracranial hemorrhage (ICH) represents a significant and life-threatening complication in pediatric patients with hemophilia [4]. The pathophysiology of hemophilia, marked by the inability to form stable blood clots, predisposes affected children to spontaneous and trauma-induced bleeding episodes, with intracranial hemorrhage being among the most severe manifestations [5].

The incidence of ICH in children with hemophilia, although relatively low, is notably higher compared to the general pediatric population. This condition necessitates immediate medical intervention due to its potential to cause irreversible neurological damage or death [6]. The presentation of ICH in these patients can vary, ranging from subtle neurological deficits to acute and catastrophic neurological deterioration [7]. Symptoms may include severe headaches, vomiting, altered consciousness, seizures, and focal neurological signs, which warrant prompt imaging studies such as computed tomography (CT) or magnetic resonance imaging (MRI) to ascertain the diagnosis [8].

Intracranial hemorrhages in children undergoing prophylaxis are reported to be quite rare. However, in children whose prophylactic treatments are not administered regularly, these life-threatening hemorrhages are more common than previously recognized [9]. Post-hemorrhagic complications may include behavioral disorders, hemiplegia/paresis, and epilepsy. Hemorrhages frequently occur following trauma and must be substantiated through imaging techniques. This condition is considered an emergency due to its high mortality and morbidity risks. In acute hemorrhage, immediate factor therapy should be initiated upon the patient’s arrival at the emergency department, with the target Factor Level being 80–100% [10].

In this study, the objective is to examine patients with hemophilia who have experienced intracranial hemorrhage, retrospectively.

## 2. Materials and Methods

### 2.1. Study Population and Participants

The data for this study were obtained from three distinct tertiary healthcare institutions (training and research hospitals, branch hospitals, and university hospitals). The study included 38 patients with Hemophilia A and Hemophilia B. Of these participants, 35 had Hemophilia A, and 3 had Hemophilia B. A total of 40 intracranial hemorrhage episodes occurring in 38 patients were evaluated.

### 2.2. Study Design

Our study aimed to share our experiences regarding intracranial hemorrhages, considered one of hemophilia’s rare yet most significant complications. This study is a multicenter, retrospective analysis using data from three tertiary care centers in a provincial city in Turkey. The demographic characteristics of the participants, particularly the age variable, were examined. All participants were male. Data were obtained from the participants’ hospital records. The presence of inhibitors against FVIII in the participants and the prophylaxis used against them were included in the analysis. Trauma history was queried, with types of traumas examined, including traffic accidents, falls, and a traumatic vaginal delivery. The duration and causes of complaints among the participants were investigated. The causes of complaints were categorized as fever, headache, convulsions, loss of consciousness, and vomiting. The participants’ Physical Examination Findings were classified as focal neurological deficits, mental status changes, speech disorders, and optic disk edemas. The duration of hospital stays was evaluated. The hemorrhage location was classified into five groups: parenchymal, subdural, scalp, subarachnoid, and multiple hemorrhagic foci. The recurrence of bleeding, the need for transfusion, surgical intervention, and mortality were also examined.

### 2.3. Measurement of Factor Levels

The participants’ Factor Levels were categorized as severe, moderate, and mild. A Factor Level of less than 1% was considered severe, 1% to 5% was considered moderate, and 5% to 45% was considered mild [11].

### 2.4. Exclusion Criteria

- Participants who refuse to participate in the study;
- Individuals with chronic inflammation;
- Those with thrombotic diseases;
- Individuals with neoplastic diseases;
- Patients who have undergone major surgical interventions in the past three months;
- Individuals who have experienced significant trauma in the past three months.

### 2.5. Examined Variables

- Age;
- Presence of inhibitors;
- Prophylaxis;
- Trauma history;
- Duration and causes of complaints;
- Results of physical examination;
- Duration of hospital stays;
- Location of hemorrhage;
- Transfusion history;
- Surgical intervention;
- Mortality rates.

### 2.6. Ethics

Ethical approval was obtained from the institutions where the research was conducted. Participation in the study was based on voluntary consent. Informed consent was obtained from all participants’ parents. All stages of the research adhered to the principles of the Declaration of Helsinki.

### 2.7. Statistical Analysis

Data were analyzed using the Statistical Package for the Social Sciences (SPSS) software version 25 (IBM Corp, Armonk, NY, USA). Descriptive statistics were provided for clinical and demographic characteristics. Chi-square and Mann–Whitney U tests were used to compare groups. Bivariate linear regression analysis was conducted to explore relationships between mortality and various participant-related variables. A *p*-value of less than 0.05 was considered statistically significant in all analyses.

## 3. Results

The average age of participants was 37.87 ± 38.16 months. Table 1 shows some characteristics of the participants. A significant difference was observed between the survival rates of participants concerning age and transfusion status in those under 36 months (see Table 2).

The oldest participant is 24 years old. All participants are male. The Factor Level is classified as mild in 1 participant, moderate in 2, and severe in 35. Inhibitor development against FVIII was identified in seven participants. Prophylaxis was administered to 23 participants.

Among the participants, 9 participants had spontaneous intracranial bleeding, 2 experienced cranial trauma as a result of traffic accidents, and 25 participants were exposed to head trauma due to falls. Of the remaining individuals, one suffered head trauma from a severe impact, and one had cranial trauma following a traumatic vaginal delivery.

In four participants, fever complaints were observed; in eight, headaches; in ten, convulsions; in six, loss of consciousness; and in ten, vomiting. It was determined that complaints persisted for a week or longer in four participants, and in thirty-four participants, complaints developed in a few days.

In ten participants, focal neurological deficits were seen; mental status change was detected in nineteen participants, speech disorders in two of them, and optic disk edemas in seven of them.

In evaluating the hospital stay, thirteen participants required hospitalization for one week, and twenty-five participants required hospitalization for longer.

Hemorrhage in the parenchyma was detected in eleven participants, and multiple hemorrhagic foci were identified in fifteen participants. Subdural hemorrhage was seen in eight participants, scalp hemorrhage in one, and subarachnoid hemorrhages were found in three participants.

Fourteen participants required transfusion, and three underwent surgical intervention.

According to the statistical analyses, the variables Factor Level, Physical Examination Findings, Transfusion, Recurrent Bleeding, Inhibitor, and Prophylaxis significantly affected survival (Table 3). No significant relationship was determined between the other analyzed variables and survival. Five of the participants examined in our study died. Accordingly, the mortality rate identified in our study is 13.1%.

This model was examined using bivariate linear regression analysis, revealing that the variables of Factor Level, Physical Examination Findings, Transfusion, Inhibitor, and Recurrent Bleeding significantly impacted survival.

According to the analysis, the presence of findings such as convulsions and loss of consciousness during admission accompanying the physical examination increases the risk of death approximately 3.5 times. (Table 4).

## 4. Discussion

Intracranial hemorrhage (ICH) incidence and severity in patients with hemophilia are closely related to the levels of circulating clotting factors. Patients with severe hemophilia are at significantly higher risk due to their nearly absent functional clotting factors, which hampers their ability to form stable hemostatic plugs after vascular injuries. ICH is a serious complication in hemophilia patients, often resulting in disability or even death. Although it can occur at any age, it is particularly common in neonates and children. In cases of hemophilic children who experience intracranial hemorrhage, severe factor deficiency is frequently identified. In one study, all hemophilic children with ICH were found to have severe factor deficiency [12]. In our study, severe factor deficiency was detected in 35 out of 38 participants, resulting in a frequency of 89%.

The initial symptoms of intracranial bleeding in children with hemophilia often include nonspecific signs, such as persistent headaches and irritability, which can be mistaken for less severe conditions [13,14]. As the hemorrhage progresses, more apparent neurological symptoms may develop. These can include altered levels of consciousness, ranging from lethargy to coma, indicating increasing intracranial pressure and cerebral involvement [15].

The pathophysiology of intracranial hemorrhage (ICH) in children with hemophilia involves a disruption of vascular integrity within the central nervous system. Due to insufficient clotting factors, the hemostatic plug formation is delayed or ineffective, leading to uncontrolled bleeding in the intracranial space. This bleeding can result from minor head trauma or occur spontaneously, with the latter often being particularly insidious because there may be no apparent precipitating event [15]. Studies have shown that fractures, such as those at the skull base and upper cervical region, which can result from head trauma, frequently lead to ICH. In our study, we examined the various conditions that contribute to cerebral hemorrhage. While 80% of the participants had a history of head trauma, eight participants experienced spontaneous cerebral hemorrhage without any history of trauma [16,17].

Intracranial bleeding is a serious and potentially fatal complication that poses significant challenges for pediatric patients diagnosed with hemophilia [4]. The immature hemostatic mechanisms in children further increase the risk of ICH, necessitating prompt and accurate medical intervention [16]. During their developmental stages, the cerebrovascular autoregulation system in children often leads to turbulent hemodynamics, which increases the likelihood of vascular rupture and predisposes them to ICH. Some studies have characterized the relationship between hypoxemia and the development of severe ICH in the context of immature cerebrovascular autoregulation [16,17]; however, other studies have not confirmed this relationship [18].

In an analysis of related studies, we found that the mortality rate was lower than that identified in our study. This discrepancy may be due to the high number of participants with severe factor deficiencies in our cohort [10]. Our findings indicate that the mortality rate is higher among participants under 36 months of age. Additionally, we observed that this age group experiences a greater frequency of transfusions, Recurrent Bleeding, and surgical interventions. Literature supporting this conclusion aligns with our results: the mortality rate in participants younger than 36 months was approximately 17%, compared to about 12% in those older than 36 months. This difference was statistically significant.

Overall, the mortality rate in our study is approximately 13%, which is significantly high. In a comparable study, the mortality rate was reported at 2.5% [19], while another study indicated that the rate was about three times higher [20]. The variations in mortality rates can be attributed to differences in the number of participants and the severity of cases examined. Other studies in the literature have reported mortality rates similar to ours [21].

Moreover, the mortality rate among participants experiencing recurrent hemorrhages was 16.6%, which was significantly higher than the rate for participants without recurrent hemorrhages in our study. The frequency of Recurrent Bleeding in similar studies was lower than we observed [22].

Clinically, intracranial bleeding can present a range of neurological symptoms, from subtle behavioral changes and irritability to severe manifestations such as seizures, focal neurological deficits, and altered consciousness [23]. Diagnosing these conditions primarily relies on neuroimaging techniques, with computed tomography (CT) and magnetic resonance imaging (MRI) playing crucial roles in determining the extent and precise location of the hemorrhage [24].

During the pediatric period, the severity of hemophilia is the most significant risk factor for intracranial hemorrhage (ICH). Andersson and colleagues investigated patients with severe hemophilia and highlighted the importance of prophylaxis in reducing the risk of ICH (20). Similarly, Bladen and colleagues studied hereditary bleeding disorders in over 1000 participants and confirmed that ICH episodes in children with hemophilia predominantly occur in those with severe disease. Another study involving 23 patients with hemophilia A and B identified critical events [25], asserting that severe disease plays a crucial role in developing ICH [9]. Our data support these findings. Our study observed that the mortality rate was lower among participants who received prophylaxis than those who did not. This led to the conclusion that the mortality rate is higher in participants receiving prophylaxis; however, this finding was statistically significant due to these patients experiencing more severe trauma and multifocal hemorrhages.

Consequently, even minor head trauma can lead to substantial hemorrhagic events [26,27]. In pediatric populations, the risk of ICH is heightened by the greater likelihood of head injuries associated with developmental milestones and physical activities [28]. Additionally, the immaturity of the pediatric neurological system intensifies the effects of ICH, potentially resulting in severe neurological deficits or death if not promptly addressed [29]. In our study, we determined that the frequency of spontaneous cerebral hemorrhage among participants was 21%, whereas the frequency of bleeding resulting from head trauma was approximately 80%.

Focal neurological deficits, such as hemiparesis or cranial nerve palsies, can develop, indicating localized areas of brain damage [30]. These deficits often correlate with the specific site of hemorrhage, providing critical diagnostic clues. Additionally, visual disturbances, including blurred vision or loss of vision, may occur due to optic nerve compression or involvement of the visual pathways [31]. In a study involving different participants, changes in mental status were the most commonly observed findings during physical examinations, while focal neurological deficits were the second most frequently identified symptoms. However, some studies recorded neurological deficits as the most prevalent finding [32]. Another notable observation was that mortality was more common among participants who exhibited changes in mental status, with a mortality rate of 21% in this group.

Inhibitors were detected in seven participants. Five of these individuals with detected inhibitors experienced Recurrent Bleeding. Long-term complications, including motor deficits and cognitive impairments, were identified in these cases. Similar studies in the literature support our findings [33]. In a study involving 112 hemophilia patients, 88 of whom presented with intracranial hemorrhage, several risk factors for intracranial hemorrhage were identified. These included an age under 3 years or over 50 years, hemophilia severity, and the presence of inhibitors [10]. The study noted that the mortality rate associated with intracranial hemorrhage was higher in patients with inhibitors, by approximately 50%.

Additionally, about 20% of the surviving patients were found to have significant disabilities. In our study, the frequency of inhibitors was 19%, and the mortality rate among patients with inhibitors was approximately 43%. The data from both studies are mutually supportive. It was also determined that the mortality rate was higher in participants who experienced bleeding from multifocal sources compared to other hemorrhagic regions, with a frequency of about 20% in this group. Despite new hemophilia treatments, the increasing number of specialized hemophilia centers, and the development of appropriate treatment strategies, intracranial hemorrhages continue to occur and remain a serious concern today.

## 5. Conclusions

ICH remains a critical complication in childhood hemophilia, demanding timely diagnosis and multidisciplinary management to mitigate morbidity and mortality. The inherent bleeding propensity in hemophilic patients underscores the necessity for vigilant clinical monitoring, particularly in the presence of minor trauma or spontaneous bleeding episodes. Advances in prophylactic factor replacement therapy and recombinant clotting factors have significantly reduced the incidence and severity of ICH, yet gaps remain in universal access and adherence to these interventions. Early recognition of neurological symptoms, prompt imaging, and factor replacement are pivotal in improving patient outcomes.

Future research should aim to refine treatment protocols, evaluate the long-term efficacy of emerging therapies, and explore novel biomarkers for early detection of intracranial bleeding. Furthermore, public health efforts must focus on enhancing awareness among caregivers and healthcare providers, especially in low-resource settings, to facilitate early intervention and reduce the burden of ICH in this vulnerable population. By integrating advances in medical science with comprehensive care strategies, the prognosis for children with hemophilia and ICH can be substantially improved, fostering a better quality of life and long-term health outcomes. ICH remains a serious complication in children with hemophilia, requiring prompt diagnosis and interdisciplinary management to reduce morbidity and mortality. Due to their inherent tendency to bleed, patients with hemophilia necessitate careful clinical monitoring, especially after minor trauma or spontaneous bleeding episodes. Advances in prophylactic factor replacement therapy and recombinant clotting factors have significantly decreased the incidence and severity of ICH. However, challenges persist in ensuring universal access to these treatments and adherence to them.

Recognizing neurological symptoms early and providing prompt imaging and factor replacement are crucial in improving patient outcomes. Future research should focus on refining treatment protocols, assessing the long-term effectiveness of new therapies, and identifying novel biomarkers for the early detection of intracranial bleeding. Additionally, public health initiatives must aim to increase awareness among caregivers and healthcare providers, particularly in low-resource settings, to enable early intervention and lessen the impact of ICH in this vulnerable population.

By integrating medical science advancements with comprehensive care strategies, we can significantly improve the prognosis for children with hemophilia and ICH, enhancing their quality of life and long-term health outcomes.

## Figures and Tables

**Table 1:** Some characteristics of the participants.

	**Mean (SD)**	
Age (Months)	37.87 ± 38.16	
	**Mild/Moderate (%)**	**Severe (%)**
Factor Level	3 (7.7)	35 (92.3)
	**Yes (%)**	**No (%)**
Transfusion	14 (36.8)	24 (63.2)
Recurrent Bleeding	8 (21)	32 (79)
Inhibitor	7 (18.4)	31 (81.6)
Prophylaxis	23 (60.5)	15 (39.5)

**Table 2:** Survival rates and the age variable among the participants.

		<36 Month	>36 Month	*p*
Transfusion	Yes	10	4	0.03
	No	11	13	
Recurrent Bleeding	Yes	6	2	0.02
	No	17	15	
Surgery	Yes	6	7	0.8
	No	13	12	
Alive/Dead	Dead	3	2	0.03
	Alive		16	

**Table 3:** Variables with a relationship to survival.

		Treated	Exitus	*p*
Factor Level	Mild/Moderate	3	0	0.01
	Severe	30	5	
Physical Examination Findings	Focal Neurological Deficit	9	1	0.02
	Mental Status Change	15	4	
	Speech Disorder	2	-	
	Optic Disk Edema	7	-	
Hemorrhagic Region	Parenchymal	10	1	0.2
	Subdural	7	1	
	Scalp	1	-	
	Subarachnoid	3	-	
	Multiple Hemorrhagic Foci	12	3	
Transfusion	Yes	11	3	0.03
	No	22	2	
Inhibitor	Yes	5	2	0.02
	No	28	3	
Recurrent Bleeding	Yes	7	1	0.01
	No	28	4	
Prophylaxis	Yes	22	1	0.03
	No	11	4	

**Table 4:** Bivariate linear regression analysis.

	B	S.E.	Wald *	df	Sig.	Exp (B) **
Factor Level	−3.307	2.312	2.045	1	0.153	0.037
Physical Examination Findings	1.258	0.698	3.246	1	0.072	3.517
Transfusion	3.019	1.694	3.176	1	0.075	2.464
Inhibitor	1.011	2.134	1.341	1	0.085	2.591
Recurrent Bleeding	3.582	1.259	1.211	1	0.359	1.781
Constant	−2.410	3.058	0.621	1	0.431	0.090

* Wald: The Wald test is used in the context of regression analysis to analyze whether the parameters in the model are significantly different from the default values. It tests the significance of the coefficients. ** Exp B, or the odds ratio, is the predicted change in odds for a unit increase in the predictor.

## Data Availability

No new data were created. Data is unavailable due to privacy or ethical restrictions.
